# Impact of Intensive Care Unit Type and Organizational Factors on Mortality in Patients Transferred from Internal Medicine Services to the Intensive Care Unit: A Prospective Cohort Study

**DOI:** 10.3390/healthcare14091206

**Published:** 2026-04-30

**Authors:** Melike Yüce Aktepe, Özlem Çakın, Özlem Esra Yıldırım, Çağlayan Merve Ayaz Ceylan, Hakan Çakın

**Affiliations:** 1 Division of Intensive Care, Department of Internal Medicine, Faculty of Medicine, Akdeniz University, 07070 Antalya, Türkiye; melikeyuceaktepe@akdeniz.edu.tr (M.Y.A.); ozlemcakin@akdeniz.edu.tr (Ö.Ç.); 2 Department of Internal Medicine, Faculty of Medicine, Akdeniz University, 07070 Antalya, Türkiye; creat.oey.07@gmail.com; 3 Department of Infectious Diseases and Clinical Microbiology, Faculty of Medicine, Akdeniz University, 07070 Antalya, Türkiye; merve.ayz@hotmail.com; 4 Department of Neurosurgery, Faculty of Medicine, Akdeniz University, 07070 Antalya, Türkiye

**Keywords:** internal medicine, intensive care unit, interhospital transfer, mortality, health services accessibility, continuity of patient care

## Abstract

**Background**: Clinical deterioration is common among patients hospitalized in Internal Medicine wards and frequently results in the need for Intensive Care Unit (ICU) admission. Limited ICU bed capacity may lead to delays or interhospital transfers, potentially affecting outcomes. This study evaluated the association between ICU follow-up location and mortality, and its relationship with organizational processes. **Methods**: In this prospective cohort study, adult patients (≥18 years) requiring ICU consultation between March 2024 and February 2025 were consecutively enrolled. Demographic characteristics, comorbidities, ICU indications, waiting times, mortality, and ICU type were recorded. Patients were categorized into three groups: Internal Medicine ICU, Anesthesiology ICU, and external ICU. The primary endpoint was overall ICU mortality. Multivariable logistic regression was performed to identify independent predictors. **Results**: A total of 331 patients were included (median age 64 years; 59.2% male). Of these, 34.7% were admitted to the Internal Medicine ICU and 24.5% to the Anesthesiology ICU, and 40.8% were transferred externally. Seven-day, 14-day, and overall ICU mortality were significantly higher in the external ICU group (all *p* < 0.001). External ICU transfer was independently associated with mortality (OR 3.26; 95% CI 1.70–6.26; *p* < 0.001), along with pre-ICU intubation and sepsis. **Conclusions**: Mortality is high among deteriorating Internal Medicine patients requiring ICU care. External ICU transfer is strongly associated with increased mortality, highlighting the potential relevance of ICU accessibility and continuity of care.

## 1. Introduction

Internal Medicine wards serve a heterogeneous patient population characterized by advanced age, multimorbidity, and high rates of immunosuppression. These features increase the risk of clinical deterioration during hospitalization and often lead to an acute and unpredictable need for Intensive Care Unit (ICU) admission [1]. In this patient group, early recognition of critical illness and timely access to appropriate ICU care are among the key prognostic determinants influencing mortality.

However, ICU beds represent one of the most limited resources in modern healthcare systems and account for only 2–8% of total hospital bed capacity in most countries [2,3]. According to the *Health at a Glance 2023* report published by the Organisation for Economic Co-operation and Development (OECD), ICU bed capacity varies substantially across member countries, with an average of approximately 17 ICU beds per 100,000 population. Despite capacity expansions in recent years, ICU beds remain a critical and constrained healthcare resource. Comparative data in the same report indicate that Türkiye has approximately 40 ICU beds per 100,000 population, exceeding the OECD average.

Nevertheless, the total number of hospital beds in Türkiye (including both ward and ICU beds) remains below the OECD average. This suggests that, within the existing bed pool, the proportion of ICU beds may be relatively high, while general ward bed capacity may be comparatively limited. Moreover, the heterogeneous distribution of ICU beds across primary, secondary, and tertiary healthcare institutions underscores that ICU accessibility and organizational structure continue to play a determining role in clinical outcomes [4]. These findings indicate that ICU capacity should not be evaluated solely in terms of absolute bed numbers but rather from a balanced and integrated healthcare planning perspective encompassing all levels of the continuum of care.

In critically ill patients, clinical characteristics alone do not determine outcomes or referral decisions. Institutional organization, resource allocation, and the management of critically ill patients outside the ICU also play significant roles. In this context, organizational factors encompass several system-level components, including ICU bed availability, the transition process from ward to ICU, inter-team patient handover, and potential disruptions in continuity of care. Previous studies have demonstrated that deteriorating ward patients frequently experience delays in ICU access, resulting in suboptimal early resuscitation and potentially increased mortality [5,6]. However, existing evidence is largely based on retrospective and observational data. Furthermore, prospective comparative data specifically addressing patients referred from Internal Medicine wards remain limited.

Importantly, few studies have prospectively evaluated the independent effect of ICU type on mortality among patients transferred from Internal Medicine wards. Therefore, the present study aimed to evaluate whether an association exists between ICU follow-up location and mortality and whether this association may be related to organizational factors after adjustment for available clinical severity indicators. We believe that this study, based on real-world prospective data, will systematically evaluate the association between ICU type, organizational factors, and outcomes in critically ill patients referred to the ICU and thereby address an important gap in the existing literature.

## 2. Materials and Methods

### 2.1. Study Design and Ethical Approval

This prospective cohort study was conducted at Akdeniz University Faculty of Medicine Hospital between March 2024 and February 2025. The total bed capacity of the in-hospital tertiary-level Internal Medicine Intensive Care Unit (ICU) and the Department of Anesthesiology and Reanimation ICU was 39 beds.

Ethical approval was obtained from the Akdeniz University Clinical Research Ethics Committee (28 March 2024; decision no. TBAEK-177). As the study was observational in nature, informed consent was not required and was not requested by the ethics committee. The study protocol was conducted in accordance with the Declaration of Helsinki.

### 2.2. Study Population

Patients aged ≥18 years who were hospitalized in Internal Medicine wards and for whom an ICU consultation was requested based on clinical deterioration were included.

Exclusion criteria were defined as refusal of ICU admission by the patient or their legal representative, incomplete follow-up data due to unavailable transfer or mortality records, and death occurring prior to physical ICU transfer despite a documented ICU admission indication. Patients were excluded when transfer or outcome information could not be reliably confirmed after ICU referral, most commonly due to incomplete documentation following interhospital transfer.

### 2.3. Study Objectives and Outcomes

The primary objective of the study was to determine ICU mortality among critically ill patients transferred from Internal Medicine wards. The primary endpoint was total ICU mortality, defined as death occurring during the ICU stay.

Secondary endpoints included early ICU mortality (7-day and 14-day mortality calculated from the date of ICU admission) and the waiting time between the documented ICU admission decision and physical ICU transfer, which was considered a key organizational determinant potentially affecting mortality.

### 2.4. Data Collection and Definitions

Patient data were recorded daily by a senior attending physician and a supervising faculty member for all patients with a documented ICU indication. Demographic characteristics (age, sex, and comorbidities), location of hospitalization prior to ICU admission, indication for ICU admission, waiting time until ICU transfer, type of ICU to which the patient was admitted, endotracheal intubation status prior to ICU admission, presence of cardiopulmonary arrest before ICU transfer, length of hospital stay prior to ICU admission, and early (7-day and 14-day) as well as total ICU mortality outcomes were systematically documented.

The indication for ICU admission was classified under a single dominant clinical category at the time of consultation, based on the primary clinical condition determined by the responsible physician.

Patients who clinically deteriorated in the ward and required ICU evaluation were initially assessed by the senior ward resident physician. ICU admission criteria were based on the Society of Critical Care Medicine (SCCM) guidelines [7]. Following this preliminary assessment, the final ICU admission decision was made through a multidisciplinary consultation involving an ICU fellow and a faculty member.

Patients were placed on a chronological waiting list according to the documented date and time of ICU indication. If a bed was available in the Internal Medicine ICU, patients were admitted preferentially to that unit. In the absence of availability, patients were considered for admission to the Anesthesiology ICU, and if no in-hospital ICU bed was available, they were transferred to an external ICU. Patients awaiting admission due to bed unavailability were registered as waiting patients. Transfers to external ICUs were determined solely by in-hospital ICU bed unavailability; no triage based on perceived prognosis, comorbidity burden, or therapeutic limitation was applied in deciding external transfer.

Waiting time for ICU admission was defined as the interval between the documented ICU indication time and the time of physical ICU admission. For patients transferred to an external ICU, waiting time was defined as the interval between the ICU admission decision and the time of admission to the receiving ICU. Chronological allocation of ICU beds was conducted in accordance with international ethical recommendations supporting chronological prioritization in the distribution of limited critical care resources [8]. For patients transferred to external ICUs, admission time was confirmed through the regional transfer coordination system, ensuring that waiting time consistently reflected the interval between ICU indication and actual admission to an ICU bed, independent of transfer location.

Transfers to external ICUs were not due to technical or clinical inadequacy of our institution but solely due to ICU bed unavailability. All external transfers were made to tertiary-level ICUs capable of providing advanced life support.

In our healthcare system, formal end-of-life care limitation decisions (including do-not-intubate orders or comfort-focused care directives) are not routinely implemented as standardized decision-making tools at the time of ICU consultation due to legal and ethical considerations. Therefore, ICU triage decisions are primarily based on acute clinical indication and real-time bed availability rather than anticipated long-term prognosis, malignancy stage, or perceived treatment futility. External ICU transfers are coordinated through a centralized regional referral system operating at the regional level. When ICU bed capacity is insufficient, transfer requests are communicated to the central coordination unit, and patient transfers are arranged according to chronological order of request rather than clinician preference or subjective assessment of prognosis. This system aims to ensure equitable access to ICU care and to minimize discretionary selection bias in transfer decisions.

Standardized illness severity scores such as SOFA and APACHE II were not routinely calculated at the time of ward-based ICU consultation because the clinical decision-making process primarily focused on rapid triage rather than structured research documentation. Retrospective reconstruction of validated severity scores was not feasible because several required physiological and laboratory parameters (e.g., arterial blood gas values, neurological scoring, and worst values within defined time windows) were not consistently available at the exact time of ICU indication.

Early mortality was defined as death occurring within 7 and 14 days after ICU admission. Total ICU mortality referred to deaths occurring during the ICU stay. Mortality data for externally transferred patients were primarily confirmed through communication with the receiving ICU teams. In a small number of cases (*n* = 5), outcome information could not be obtained directly from institutional records and was therefore confirmed through contact with family members.

### 2.5. Group Classification

Patients were categorized into three groups according to the ICU to which they were admitted:

(1) Patients admitted to the in-hospital Internal Medicine ICU;
(2) Patients admitted to the in-hospital Anesthesiology ICU;
(3) Patients transferred to an external ICU.

In the primary analysis, demographic characteristics, clinical variables, and mortality outcomes were compared across these three groups.

In the secondary analysis, all patients admitted to in-hospital ICUs were analyzed collectively as the hospital-based ICU group and compared with patients transferred to an external ICU (external ICU group) to evaluate more clearly the impact of ICU follow-up location on mortality.

Additionally, patients were analyzed according to survival status (survivors vs. non-survivors).

### 2.6. Statistical Analysis

Statistical analyses were performed using IBM SPSS Statistics software (Version 24.0; IBM Corp., Armonk, NY, USA).

The distribution of continuous variables was assessed using visual methods (histograms and probability plots) and analytical methods (Kolmogorov–Smirnov test). Continuous variables were expressed as mean ± standard deviation (SD) or median with interquartile range (IQR), as appropriate, whereas categorical variables were presented as frequencies and percentages.

Comparisons between two groups were performed using Student’s *t* test for normally distributed variables and the Mann–Whitney U test for non-normally distributed variables. For comparisons involving three or more groups, one-way analysis of variance (ANOVA) was used for normally distributed continuous variables, and the Kruskal–Wallis test was used for non-normally distributed variables. When a statistically significant difference was detected, appropriate post-hoc pairwise comparisons were performed using Bonferroni correction. Categorical variables were compared using the chi-square test or Fisher’s exact test, as appropriate.

To identify independent predictors of ICU mortality, multivariable logistic regression analyses were conducted. Variables associated with ICU mortality at *p* < 0.25 in univariable analyses, together with clinically relevant variables, were included in the multivariable models. In the sensitivity analysis, a simplified multivariable model was constructed to evaluate the robustness of the primary association while reducing the risk of model overfitting. Variables with multiple categories and small subgroup sizes, such as consulting division, were not included in the simplified model in order to preserve model stability and obtain more reliable parameter estimates.

An additional sensitivity analysis restricted to patients without malignancy was performed to evaluate whether the association between external ICU transfer and mortality persisted after exclusion of oncology patients.

Results were reported as odds ratios (ORs) with 95% confidence intervals (CIs). Model calibration was assessed using the Hosmer–Lemeshow goodness-of-fit test and model discrimination was assessed using the C-statistic. A two-sided *p* value < 0.05 was considered statistically significant.

## 3. Results

### 3.1. Study Population and Baseline Characteristics

During the study period, a total of 448 patients hospitalized in Internal Medicine wards for whom an ICU consultation was requested were assessed for eligibility. Of these, 117 patients met the exclusion criteria and were excluded. Consequently, 331 patients were prospectively included in the final analysis.

**Figure 1:**
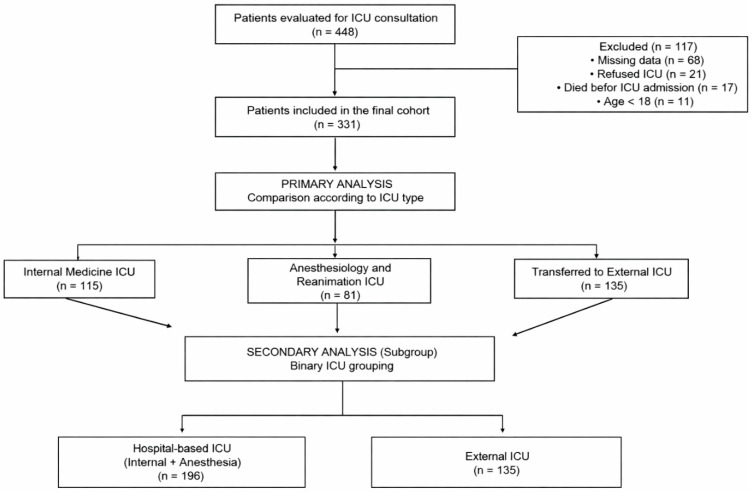
Flow diagram of patient admissions.

In the primary analysis, critically ill patients were compared across three groups according to ICU follow-up location. In the secondary analysis, 196 patients were followed in in-hospital ICUs, while 135 patients were transferred to an external ICU (Figure 1).

The median age of the study population was 64 years (interquartile range [IQR], 56.0–73.5), and 59.2% were male. Although the distribution of consulting divisions differed significantly between mortality groups, oncology was the most frequent department requesting ICU consultation (49.8%), followed by hematology (17.2%) and gastroenterology (14.8%) (*p* = 0.004).

Overall, 34.7% of patients were admitted to the Internal Medicine ICU and 24.5% to the Anesthesiology ICU, and 40.8% were transferred to an external ICU. A statistically significant association was observed between ICU follow-up location and mortality, with higher mortality rates among patients transferred to external ICUs (*p* < 0.001).

When ICU admission indications were compared according to survival status, sepsis was more frequent among non-survivors than survivors (78.6% vs. 66.3%, *p* = 0.02), whereas respiratory failure was more common among survivors (15.7% vs. 7.3%, *p* = 0.02). Compared with survivors, non-survivors more frequently required endotracheal intubation prior to ICU admission (43.5% vs. 15.7%, *p* < 0.001), had a history of cardiopulmonary arrest (7.7% vs. 1.2%, *p* = 0.03), and had longer hospital stays prior to ICU admission (*p* = 0.003).

The median waiting time for ICU admission was 4.5 h (IQR, 2.0–12.0), and no statistically significant difference was observed between groups (*p* = 0.23) (Table 1).

### 3.2. Comparison According to ICU Follow-Up Location

When groups were compared according to ICU follow-up location, patients transferred to an external ICU were older than those admitted to in-hospital ICUs (median age: 67.0 vs. 64.0 vs. 63.0 years, respectively; *p* = 0.007).

The distribution of consulting divisions differed significantly across ICU types (*p* = 0.001). Oncology patients were more frequently transferred to external ICUs (*n* = 88), whereas hematology patients were more commonly admitted to the Internal Medicine ICU (*n* = 28).

ICU admission indications, including respiratory failure, gastrointestinal symptoms, and sepsis, were similar across groups (*p* = 0.65, *p* = 0.39, and *p* = 0.18, respectively). There were no statistically significant differences among groups regarding pre-ICU intubation requirement, history of cardiopulmonary arrest, or waiting time until ICU transfer (*p* = 0.08, *p* = 0.14, and *p* = 0.12, respectively).

Early ICU mortality was significantly higher in the external ICU group compared with in-hospital ICU groups. In total, 7-day mortality rates were 57.8%, 46.9%, and 33.0%, respectively (*p* < 0.001), and 14-day mortality rates were 78.5%, 65.4%, and 50.4%, respectively (*p* < 0.001).

Similarly, overall ICU mortality was significantly higher among patients transferred to external ICUs compared with those admitted to Internal Medicine and Anesthesiology ICUs (86.7% vs. 74.1% vs. 61.7%, respectively; *p* < 0.001) (Table 2).

Importantly, the distribution of major clinical instability markers, including pre-ICU intubation, cardiopulmonary arrest, and sepsis, did not differ significantly across ICU types.

### 3.3. Multivariable Analysis of ICU Mortality

To identify independent predictors of ICU mortality, multivariable logistic regression analysis was performed. Transfer to an external ICU demonstrated the strongest independent association with mortality (odds ratio [OR] 3.26; 95% confidence interval [CI] 1.70–6.26; *p* < 0.001).

Pre-ICU endotracheal intubation requirement was also independently associated with mortality (OR 3.65; 95% CI 1.74–7.68; *p* = 0.001). In addition, sepsis as the indication for ICU admission was significantly associated with increased mortality (OR 2.10; 95% CI 1.08–4.08; *p* = 0.03).

In contrast, age, sex, history of cardiopulmonary arrest prior to ICU admission, respiratory failure as an admission indication, and waiting time for ICU admission were not independently associated with mortality (*p* = 0.57, *p* = 0.29, *p* = 0.23, *p* = 0.81, and *p* = 0.09, respectively).

In analyses evaluating consulting divisions, a statistically significant association was observed for endocrinology consultation; however, this finding is based on a very small subgroup and should be interpreted cautiously.

Model calibration assessed using the Hosmer–Lemeshow goodness-of-fit test indicated good agreement between observed and predicted outcomes (*p* = 0.671) (Table 3).

### 3.4. Sensitivity Analysis

A simplified multivariable logistic regression model was constructed as part of the sensitivity analysis. In this model, pre-ICU endotracheal intubation remained independently associated with ICU mortality (OR 3.26; 95% CI 1.62–6.53; *p* = 0.001).

Similarly, transfer to an external ICU remained a strong and independent predictor of mortality (OR 3.70; 95% CI 2.00–6.83; *p* < 0.001).

Age, sex, history of cardiopulmonary arrest, respiratory failure indication, presence of sepsis, and waiting time for ICU admission were not independently associated with mortality. Model calibration remained acceptable (Hosmer–Lemeshow *p* = 0.95), supporting the robustness of the primary findings (Table 4).

To further evaluate the potential impact of case-mix differences related to malignancy status, a sensitivity analysis restricted to patients without malignancy was performed. In this subgroup analysis, transfer to an external ICU remained independently associated with ICU mortality (OR 3.94, 95% CI 1.48–10.53, *p* = 0.02) (Supplementary Table S1).

## 4. Discussion

This prospective cohort study demonstrates that mortality among patients transferred to the Intensive Care Unit (ICU) following clinical deterioration in Internal Medicine wards is high and varies significantly according to ICU follow-up location. The primary endpoint, total ICU mortality, was markedly higher among patients transferred to external ICUs compared with those admitted to in-hospital ICUs. Similarly, 7-day and 14-day mortality were significantly increased in the external ICU group, highlighting the early post-transfer period as a particularly vulnerable clinical phase. In contrast, waiting time for ICU admission did not differ between groups and was not independently associated with mortality. Multivariable analyses consistently identified transfer to an external ICU as the strongest and most robust independent predictor of mortality. Pre-ICU endotracheal intubation and the presence of sepsis were also independently associated with mortality. Adequate model calibration further supports the statistical reliability of these findings. Collectively, these results suggest that mortality risk in critically ill patients may be associated not only with physiological disease severity but also with organizational characteristics and ICU accessibility.

The cohort was characterized by advanced age, substantial multimorbidity, and a high burden of malignancy, representing a clinically fragile population [9]. Previous studies have demonstrated increased ICU mortality among patients with malignancy and immunosuppression, largely driven by infection risk, organ dysfunction, and underlying disease burden [10,11]. Multidisciplinary ICU models have been shown to improve outcomes in this population [11]. Thus, the elevated mortality observed in our study must be interpreted within the context of a high-risk internal medicine population.

Sepsis was the most frequent indication for ICU admission. As a time-sensitive condition, sepsis outcomes depend heavily on early critical care support and efficient patient flow management [12,13,14]. However, the similar distribution of sepsis across groups indicates that admission indication alone does not explain the mortality difference. This finding strengthens the hypothesis that system-level and organizational factors may be associated with differences in outcomes.

When ICU types were compared, externally transferred patients were older and more frequently represented oncology services, whereas hematology patients were more commonly managed in the Internal Medicine ICU. Hematology patients represent a highly specialized group requiring expertise in profound immunosuppression, cytopenias, complex chemotherapy protocols, and invasive infections [11,15]. Management within experienced multidisciplinary teams may partially mitigate risk. Importantly, age was not independently associated with mortality in multivariable models, suggesting that chronological age alone does not account for the observed differences and that structural determinants may be more strongly associated with observed outcome differences. The higher proportion of oncology patients in the external ICU group may raise concern regarding potential confounding related to disease trajectory, functional status, or unmeasured treatment limitation preferences. However, in our clinical setting, treatment limitation directives are not routinely used as formal triage criteria, and ICU admission decisions are based primarily on acute clinical indication and bed availability. Therefore, external transfer decisions were not guided by predefined prognostic restriction policies.

The higher proportion of oncology patients in the external ICU group may introduce residual confounding related to disease trajectory, performance status, frailty, and treatment intent, which were not systematically captured. Although malignancy variables were not independently associated with mortality in adjusted models, the absence of detailed oncological staging and functional status indicators limits the ability to fully exclude baseline prognostic imbalance. Therefore, the observed relationship should be interpreted as an association rather than evidence of a causal effect of organizational factors on mortality.

Importantly, the association between external ICU transfer and ICU mortality remained consistent in a sensitivity analysis restricted to patients without malignancy, suggesting that differences in malignancy distribution alone are unlikely to fully explain the observed association.

The observed association between endocrinology consultation and lower mortality should be interpreted with caution due to the small number of patients in this subgroup and the wide confidence interval of the effect estimate. This finding is likely hypothesis-generating and may reflect sample variability rather than a true clinical effect. Therefore, no causal or clinically generalizable inference should be drawn from this result.

A notable finding is that mortality remained significantly higher in externally transferred patients despite comparable distributions of key severity surrogates—including pre-ICU intubation, cardiopulmonary arrest, and sepsis—as well as similar waiting times across groups. A potential concern is reverse causality, whereby more unstable patients may have been preferentially transferred externally to preserve in-hospital ICU capacity. However, the absence of significant differences in baseline instability markers and waiting times across ICU types argues against substantial systematic severity-driven transfer bias. Although pre-ICU intubation was independently associated with mortality, transfer to an external ICU remained a strong predictor even after multivariable adjustment. These findings support the interpretation that organizational factors—such as continuity of care, coordination between teams, and fragmentation of treatment processes—may be associated with differences in outcomes [16,17,18].

Although residual confounding cannot be completely excluded, the magnitude and consistency of the observed association across adjusted and sensitivity models suggest that baseline severity differences alone are unlikely to fully explain the findings. Therefore, the persistence of an odds ratio exceeding 3 across both primary and sensitivity analyses suggests that residual imbalance in baseline severity alone is unlikely to fully account for the observed association between external ICU transfer and mortality.

Therefore, residual confounding related to unmeasured differences in baseline severity cannot be entirely excluded. The observation that unmeasured confounding in observational studies often biases estimates toward the null should be interpreted as a heuristic consideration rather than a definitive methodological justification. Accordingly, the present findings should be interpreted as demonstrating an association between external ICU transfer and mortality rather than a causal effect. Future multicenter studies incorporating standardized severity scores at the time of ICU consultation are needed to further clarify the relative contribution of clinical and organizational determinants of mortality.

Importantly, all interhospital transfers were conducted through the national emergency medical service using advanced life support ambulances, and no deaths occurred during transport. Furthermore, all receiving centers were tertiary-level ICUs with comparable technical capacity. Therefore, the increased mortality cannot reasonably be attributed to transport-related complications or differences in care level. Prior literature suggests that increased mortality among transferred patients may be related to pre-transfer stabilization adequacy, communication gaps between teams, and disruptions in post-transfer care continuity [19,20]. Transitional phases, including re-evaluation of treatment plans and changes in care teams, may contribute to early mortality vulnerability.

These findings underscore the need for a comprehensive interhospital ICU transfer framework that extends beyond transport safety to include decision-making processes, bed management strategies, timing optimization, and preservation of care continuity. The markedly elevated mortality among externally transferred patients also raises important ethical considerations regarding equitable ICU resource allocation. Although chronological prioritization has been recommended under conditions of limited ICU capacity [8], heterogeneity in clinical urgency and inter-center variability may limit the effectiveness of a purely time-based allocation strategy. Mortality should therefore be conceptualized as a multidimensional outcome influenced by clinical severity, healthcare system organization, and timing of intervention.

The slightly higher odds ratio observed in the sensitivity model likely reflects differences in covariate inclusion rather than a true change in effect size. Importantly, the direction, magnitude, and statistical significance of the association between external ICU transfer and mortality remained consistent across models, supporting the robustness of the primary finding.

Interestingly, waiting time for ICU admission was not independently associated with mortality. In our setting, transfers are centrally coordinated by a regional command system, allowing rapid placement decisions based on institutional capacity without reliance on individual physician acceptance processes. This centralized structure may explain the absence of a mortality association. Previous studies have reported heterogeneous results: while prolonged delays have been associated with increased mortality [15,21], shorter delays have not consistently demonstrated an independent effect [22]. The relatively short waiting times observed in our cohort align with this latter body of evidence.

From a clinical and policy perspective, our findings suggest that high-risk Internal Medicine patients may benefit from being managed, whenever feasible, within the same institution in a structured and multidisciplinary Internal Medicine ICU. Reducing the need for external transfer through optimized bed planning, strengthened triage algorithms, and standardized ward-to-ICU transition pathways may represent effective system-level interventions. Such strategies may improve not only survival but also healthcare efficiency and patient safety.

### Strengths and Limitations

This study has several important strengths. First, the prospective design and inclusion of consecutive patients experiencing clinical deterioration in Internal Medicine wards reduce selection bias and enhance internal validity. Second, the detailed evaluation of ICU bed unavailability and interhospital transfer dynamics provides valuable insight into capacity management and organizational decision-making processes. Prospective comparative data examining ICU type, transfer dynamics, and outcomes specifically within Internal Medicine populations remain limited; therefore, this study contributes novel evidence, particularly regarding mortality differences according to ICU follow-up location.

Several limitations should also be acknowledged. The single-center design may limit generalizability to institutions with different resource levels or patient profiles. However, in our region, external transfers were performed solely due to ICU bed unavailability rather than technical inadequacy, and all transfers were made to tertiary-level ICUs, reducing variability in care level. Detailed post-transfer management data from receiving centers were unavailable, limiting comprehensive evaluation of subsequent ICU care. Additionally, long-term functional outcomes and quality-of-life measures were not assessed.

Standardized illness severity scores such as SOFA and APACHE II were not routinely recorded in ward settings and could not be reliably reconstructed. Although validated severity scores were unavailable, clinically meaningful surrogate markers of baseline instability—including pre-ICU intubation, cardiopulmonary arrest, and sepsis—were systematically captured and incorporated into multivariable models. While these surrogates cannot fully replace standardized scoring systems, they partially mitigate residual confounding by severity in a ward-based cohort. The inability to compare baseline organ dysfunction severity across groups represents a methodological limitation.

Despite these limitations, the real-world prospective design enhances external validity and supports the relevance of these findings for tertiary care centers with similar patient populations and resource constraints.

The use of surrogate severity indicators may not fully capture the multidimensional physiological derangements reflected by validated scoring systems. Therefore, residual confounding by disease severity cannot be entirely excluded. However, the prospective collection of key instability markers and the consistency of effect estimates across multivariable and sensitivity models strengthens confidence in the findings. Future multicenter studies incorporating standardized severity scores at the time of ICU consultation are warranted to further clarify the relative contributions of clinical and organizational determinants of mortality.

The relatively high prevalence of malignancy in the cohort reflects the real-world case mix of a tertiary Internal Medicine service. Although malignancy variables were not independently associated with mortality in adjusted models, detailed oncologic staging, performance status, and frailty measures were not systematically available. Additionally, the absence of standardized documentation regarding treatment limitation preferences may introduce residual heterogeneity in baseline prognosis. Therefore, the high overall mortality observed in this cohort should be interpreted within the context of a severely ill population with substantial comorbidity burden.

Post-ICU outcomes such as hospital mortality and total length of stay could not be consistently obtained for patients transferred to external ICUs due to variability in institutional data accessibility and follow-up completeness. To avoid introducing differential information bias between ICU groups, these outcomes were not included in the analysis. Waiting time was defined as the interval between the documented ICU indication and physical admission to an ICU bed for all patients. However, for patients transferred to external ICUs, this interval inherently includes additional processes related to interhospital coordination and transport logistics. Although the centralized transfer system aims to standardize patient flow, the inclusion of transport-related time components may introduce definitional asymmetry between in-hospital and external ICU groups. Therefore, direct comparisons of waiting time across groups should be interpreted with caution.

## 5. Conclusions

This prospective cohort study demonstrates that mortality is high among patients who develop a need for Intensive Care Unit (ICU) admission due to clinical deterioration while hospitalized in Internal Medicine wards. Mortality rates were significantly higher among patients transferred to external ICUs compared with those admitted to in-hospital ICUs.

These findings suggest that clinical outcomes in critically ill Internal Medicine patients may be associated not only with physiological disease severity but also with ICU accessibility, organizational structure, and continuity of care. Although no significant association was observed between waiting time for ICU admission and mortality, the relatively short waiting times in this cohort may partially explain this finding.

From a clinical and health system perspective, the management of high-risk, elderly, and multimorbid patients—whenever feasible—within the same institution in structured, multidisciplinary Internal Medicine ICUs may be associated with improved outcomes. Reducing the need for external transfer, optimizing ICU bed allocation strategies, and strengthening patient flow and transition processes represent potentially modifiable system-level interventions that may help reduce mortality in this vulnerable population.

## Figures and Tables

**Table 1:** Baseline characteristics of the study population.

Characteristics	Total	Non-Survivors (*n* = 248, 74.9%)	Survivors (*n* = 83, 25.1%)	*p*-Value
**Age, years**	64.0 (56.0–73.5)	64.0 (56.0–74.0)	65.0 (59.0–72.0)	0.920
**Gender, male**	196 (59.2)	145 (58.5)	51 (61.4)	0.630
**Division asking for consultation**				**0.004**
Oncology	165 (49.8)	136 **^a^** (54.8)	29 **^b^** (34.9)	
Hematology	57 (17.2)	40 (16.1)	17 (20.5)	
Gastroenterology	49 (14.8)	36 (14.5)	13 (15.7)	
Others	60 (18)	36 (14.4)	24 (28.9)	
**Co-morbidities**				
Solid organ malignancy	177 (53.5)	144 (58.1)	33 (39.8)	**0.004**
Hypertension	97 (29.3)	64 (25.8)	33 (39.8)	**0.020**
Diabetes Mellitus	82 (24.8)	59 (23.8)	23 (27.7)	0.470
Hematologic malignancy	54 (16.3)	40 (16.1)	14 (16.9)	0.880
Pulmonary diseases	41 (12.4)	31 (12.5)	10 (12.0)	0.910
**Multimorbidity** (≥2 chronic conditions)	173 (52.3)	128 (51.6)	45 (54.2)	0.680
**Indications for ICU admission**				
Sepsis	250 (75.5)	195 (78.6)	55 (66.3)	**0.020**
Cerebrovascular accident	10 (3.0)	7 (2.8)	3 (3.6)	0.720
Hepatic failure	13 (3.9)	11 (4.4)	2 (2.4)	0.530
Shock	18 (5.4)	11 (4.4)	7 (8.4)	0.170
Respiratory failure	31 (9.4)	18 (7.3)	13 (15.7)	**0.020**
Acute decompensated HF	11 (3.3)	7 (2.8)	4 (4.8)	0.480
Status epilepticus	7 (2.1)	4 (1.6)	3 (3.6)	0.370
**ICU follow-up location**				**<0.001**
Internal Medicine	115 (34.7)	71 ^a^ (28.6)	44 ^b^ (53.0)	
Anesthesiology	81 (24.5)	60 (24.2)	21 (25.3)	
Transferred to an External ICU	135 (40.8)	117 ^a^ (47.2)	18 ^b^ (21.7)	
**Before ICU**				
Intubation	121 (36.6)	108 (43.5)	13 (15.7)	**<0.001**
Cardiopulmonary arrest	20 (6.0)	19 (7.7)	1 (1.2)	**0.030**
Length of hospital stay, days	10.0 (4.0–20.0)	11.0 (5.0–21.0)	5.0 (3.0–16.5)	0.003
Waiting time, hours	4.5 (2.0–12.0)	5.0 (2.5–13.0)	4.0 (2.0–9.0)	0.230

Values are presented as median (IQR) or *n* (%). Intensive care indications were classified under a single category based on the predominant clinical presentation at the time of consultation. a and b are used to indicate which subgroups accounted for the statistically significant difference that emerged. *p* < 0.05 was considered statistically significant. HF, heart failure; ICU, intensive care unit.

**Table 2:** Comparison of demographic and clinical characteristics of the study population according to intensive care unit type.

	Anesthesiology and Reanimation ICU (*n* = 81, 24.5%)	Transferred to an External ICU (*n* = 135, 40.8%)	Internal Medicine ICU (*n* = 115, 34.7%)	*p*-Value
**Age, years**	64.0 ^a,b^ (56.0–73.0)	67.0 ^a^ (59.0–77.0)	63.0 ^b^ (53.5–70.5)	**0.007**
**Gender**				0.270
Male	42 (51.9)	85 (63.0)	69 (60.0)	
Female	39 (48.1)	50 (37.0)	46 (40.0)	
**Division asking for consultation**				**0.001**
Internal Medicine	3 (3.7)	8 (5.9)	10 (8.7)	
Endocrinology	4 (4.9)	4 (3.0)	6 (5.2)	
Hematology	16 ^a,b^ (19.8)	13 ^a^ (9.6)	28 ^b^ (24.3)	
Oncology	36 ^a^ (44.4)	88 ^b^ (65.2)	41 ^a^ (35.7)	
Nephrology	6 (7.4)	3 (2.2)	8 (7.0)	
Rheumatology	5 (6.2)	1 (0.7)	2 (1.7)	
Gastroenterology	11 (13.6)	18 (13.3)	20 (17.4)	
Multimorbidity (≥2 chronic conditions)	38 (46.9)	65 (48.1)	70 (60.9)	0.070
**Indication for ICU admission**				
Sepsis	55 (67.9)	106 (78.5)	89 (77.4)	0.180
**Before ICU**				
Intubation	35 (43.2)	53 (39.8)	33 (28.7)	0.080
Cardiopulmonary arrest	6 (7.4)	4 (3.0)	10 (8.7)	0.140
**Mortality**				
7-day mortality	38 ^a,b^ (46.9)	78 ^a^ (57.8)	38 ^b^ (33.0)	**<0.001**
14-day mortality	53 ^a,b^ (65.4)	106 ^a^ (78.5)	58 ^b^ (50.4)	**<0.001**
ICU mortality (overall)	60 (74.1)	117 (86.7)	71 (61.7)	**<0.001**
Survivors (overall)	21 (25.9)	18 (13.3)	44 (38.3)	
**Waiting time, hours**	4.5 (2.0–12.0)	4.0 (2.0–9.0)	6.0 (3.0–14.4)	0.120

Values are presented as median (IQR) or *n* (%). a and b are used to indicate which subgroups accounted for the statistically significant difference that emerged. *p* < 0.05 was considered statistically significant. ICU, intensive care unit.

**Table 3:** Multivariable Logistic Regression Analysis Identifying Independent Predictors of ICU Mortality.

	Odds Ratio	95% Confidence Interval	*p*-Value
**Age (per one year)**	0.99	0.97–1.02	0.570
**Gender, male**	0.73	0.40–1.31	0.290
**Division asking for consultation**			
Internal Medicine	Reference		
Endocrinology	0.13	0.02–0.72	0.020
Hematology	0.93	0.26–3.25	0.900
Oncology	1.50	0.47–4.77	0.490
Nephrology	0.58	0.13–2.73	0.490
Rheumatology	1.93	0.27–13.73	0.510
Gastroenterology	1.26	0.36–4.39	0.720
**Before ICU**			
Intubation	3.65	1.74–7.68	**0.001**
Cardiopulmonary arrest	3.70	0.43–31.71	0.230
**Transferred to an External ICU**	3.26	1.70–6.26	**<0.001**
**Indications for ICU admission**			
Respiratory failure	1.08	0.58–2.03	0.810
Sepsis/Septic shock	2.10	1.08–4.08	**0.030**
**Waiting time for ICU (one per hour)**	1.02	1.00–1.04	0.090

*p* < 0.05 was considered statistically significant. ICU, intensive care unit (Hosmer–Lemeshow test *p* = 0.671; C-statistic: 0.778, 95% CI 0.719–0.836, *p* < 0.001).

**Table 4:** Final multivariable logistic regression model for ICU mortality (external ICU transfer vs hospital-based ICU).

	Odds Ratio	95% Confidence Interval	*p*-Value
**Age (per one year)**	0.99	0.97–1.01	0.510
**Gender, male**	0.73	0.42–1.28	0.280
**Before ICU**			
Intubation	3.26	1.62–6.53	**<0.001**
Cardiopulmonary arrest	4.03	0.47–34.22	0.200
**Transferred to an External ICU**	3.70	2.00–6.83	**<0.001**
**Indications for ICU admission**			
Respiratory failure	1.39	0.78–2.46	0.270
Sepsis/Septic shock	1.75	0.95–3.24	0.080
**Waiting time for ICU (one per hour)**	1.02	1.00–1.04	0.130

*p* < 0.05 was considered statistically significant. ICU, intensive care unit (Hosmer–Lemeshow test *p* = 0.954; C-statistic: 0.757, 95% CI 0.697–0.817, *p* < 0.001).

## Data Availability

The datasets generated and analyzed during the current study are not publicly available due to institutional and ethical restrictions regarding patient confidentiality but are available from the corresponding author on reasonable request, subject to approval by the institutional ethics committee.

## Supplementary Materials

The following supporting information can be downloaded at: https://www.mdpi.com/article/10.3390/healthcare14091206/s1, Table S1. Sensitivity analysis: multivariable logistic regression model for ICU mortality excluding patients with malignancy (external ICU transfer vs. hospital-based ICU).

## Abbreviations

ICU	Intensive Care Unit
OR	Odds Ratio
CI	Confidence Interval
IQR	Interquartile Range
SD	Standard Deviation
SCCM	Society of Critical Care Medicine
OECD	Organisation for Economic Co-operation and Development

## References

[1] Inci K., Aygencel G., Turkoglu M., Mercan A., Boyaci Dundar N. (2024). The Impact of Admission Timing on Intensive Care Unit (ICU) Outcomes for Patients Transferred from Internal Medicine Wards—A Single Center Study. J. Crit. Intensive Care.

[2] Wunsch H., Angus D.C., Harrison D.A., Collange O., Fowler R., Hoste E.A., de Keizer N.F., Kersten A., Linde-Zwirble W.T., Sandiumenge A. (2011). Variation in critical care services across North America and Western Europe. JAMA.

[3] Rhodes A., Ferdinande P., Flaatten H., Guidet B., Metnitz P.G., Moreno R.P. (2012). The variability of critical care bed numbers in Europe. Intensive Care Med..

[4] Organisation for Economic Co-Operation and Development (OECD) (2023). Health at a Glance 2023: OECD Indicators.

[5] Dünser M.W., Singer M., Taylor M. (2024). Emergency critical care: Closing the gap between onset of critical illness and intensive care unit admission. Wien. Klin. Wochenschr..

[6] Brown A., Ballal A., Al-Haddad M. (2021). Recognising and responding to the critically ill patient. Anaesth. Intensive Care Med..

[7] Society of Critical Care Medicine (1999). Guidelines for ICU admission, discharge and triage. Crit. Care Med..

[8] American Thoracic Society Bioethics Task Force (1997). Fair allocation of intensive care unit resources. Am. J. Respir. Crit. Care Med..

[9] Flaatten H., De Lange D.W., Artigas A., Bin D., Moreno R., Christensen S., Joynt G.M., Bagshaw S.M., Sprung C.L., Benoit D. (2017). The status of intensive care medicine research and a future agenda for very old patients in the ICU. Intensive Care Med..

[10] Azoulay É., Pène F., Darmon M., Lengline E., Benoit D., Soares M., Vincent F., Bruneel F., Perez P., Lemiale V. (2015). Managing critically ill hematology patients: Time to think differently. Blood Rev..

[11] Azoulay É., Soares M. (2020). Immune dysfunction and outcomes in critically ill cancer patients: The role of a multidisciplinary ICU model. Lancet Respir. Med..

[12] Pires H.H.G., Neves F.F., Pazin-Filho A. (2019). Triage and flow management in sepsis. Int. J. Emerg. Med..

[13] Singer M., Deutschman C.S., Seymour C.W., Shankar-Hari M., Annane D., Bauer M., Bellomo R., Bernard G.R., Chiche J.-D., Coopersmith C.M. (2016). The third international consensus definitions for sepsis and septic shock (Sepsis-3). JAMA.

[14] Ahn Y.H., Lee J., Oh D.K., Lee S.Y., Park M.H., Lee H., Lim C.M., Lee S.M., Lee H.Y., Korean Sepsis Alliance (KSA) Investigators (2023). Association between the timing of ICU admission and mortality in patients with hospital-onset sepsis: A nationwide prospective cohort study. J. Intensive Care.

[15] Kiekkas P., Tzenalis A., Gklava V., Stefanopoulos N., Voyagis G., Aretha D. (2022). Delayed admission to the intensive care unit and mortality of critically ill adults: Systematic review and meta-analysis. Biomed. Res. Int..

[16] Pronovost P.J., Angus D.C., Dorman T., Robinson K.A., Dremsizov T.T., Young T.L. (2002). Physician staffing patterns and clinical outcomes in critically ill patients: A systematic review and meta-analysis. JAMA.

[17] Pronovost P.J., Jenckes M.W., Dorman T., Garrett E., Breslow M.J., Rosenfeld B.A., Lipsett P.A., Bass E. (1999). Organizational characteristics of intensive care units related to outcomes of abdominal aortic surgery. JAMA.

[18] Nates J.L., Nunnally M., Kleinpell R., Blosser S., Goldner J., Birriel B., Fowler C.S., Byrum D., Miles W.S., Bailey H. (2016). ICU admission, discharge, and triage guidelines: A framework to enhance clinical operations, development of institutional policies, and further research. Crit. Care Med..

[19] Edmondson M.E., Reimer A.P. (2024). Outcomes after interhospital critical care transfer. Air Med. J..

[20] Kahn J.M., Rubenfeld G.D., Rohrbach J., Fuchs B.D. (2007). Association between hospital transfer and mortality among critically ill patients. Crit. Care Med..

[21] Cardoso L.T.Q., Grion C.M.C., Matsuo T., Anami E.H., Kauss I.A.M., Seko L., Bonametti A.M. (2011). Impact of delayed admission to intensive care units on mortality of critically ill patients: A cohort study. Crit. Care.

[22] Ofoma U.R., Basnet S., Berger A., Kirchner H.L., Girod J.P. (2020). Trends in survival after intensive care unit admission: Timing of admission and outcomes. J. Crit. Care.

