# Supplementary material for: Impact of Intensive Care Unit Type and Organizational Factors on Mortality in Patients Transferred from Internal Medicine Services to the Intensive Care Unit: A Prospective Cohort Study
Source: Healthcare (Basel). 2026 Apr 30;14(9):1206. doi: 10.3390/healthcare14091206 (PMC13163495; doi:10.3390/healthcare14091206)
Supplement: Supplementary file 1 [file healthcare-14-01206-s001.zip › healthcare-4199074-supplementary.pdf]

Supplementary Table S1. Sensitivity analysis: multivariable logistic regression model for ICU mortality excluding patients with malignancy (external ICU transfer vs hospital-based ICU)

	**Odds Ratio**	**95% Confidence Interval**	**p-value**
**Age (per one year)**	1.01	0.98-1.04	0.50
**Gender, male**	0.82	0.39-1.73	0.60
**Before ICU**			
Intubation	3.22	1.17-8.89	**0.02**
Cardiopulmonary arrest	2.66	0.28-25.47	0.40
**Transferred to an External ICU**	3.94	1.48-10.53	**0.02**
**Waiting time for ICU (one per hour)**	1.01	0.99-1.04	0.32

$p < 0.05$ was considered statistically significant. ICU, intensive care unit. (Hosmer-Lemeshow test p=0.799; C-statistic: 0.751, 95% CI 0.671-0.832, p<0.001)
Variables with $p < 0.20$ in univariable analysis were included in the multivariable logistic regression model.
